# Constraint-Based Sound-Motion Objects in Music Performance

**DOI:** 10.3389/fpsyg.2021.732729

**Published:** 2021-12-21

**Authors:** Rolf Inge Godøy

**Affiliations:** ^1^Department of Musicology, University of Oslo, Oslo, Norway; ^2^RITMO Centre for Interdisciplinary Studies in Rhythm, Time and Motion, University of Oslo, Oslo, Norway

**Keywords:** sound objects, performance, intermittency, motor gestalt, constraints

## Abstract

The aim of this paper is to present principles of constraint-based sound-motion objects in music performance. *Sound-motion objects* are multimodal fragments of combined sound and sound-producing body motion, usually in the duration range of just a few seconds, and conceived, produced, and perceived as intrinsically coherent units. Sound-motion objects have a privileged role as building blocks in music because of their duration, coherence, and salient features and emerge from combined instrumental, biomechanical, and motor control constraints at work in performance. Exploring these constraints and the crucial role of the sound-motion objects can enhance our understanding of generative processes in music and have practical applications in performance, improvisation, and composition.

## Introduction

### Object Perspectives

A rapid harp glissando, a fast drum fill, a harpsicord ornament, a trumpet signal, or a tremolo crescendo on a cymbal are all examples of sound-motion objects in the sense that they combine salient sound energy envelopes with salient sound-producing body motion shapes into strongly coherent entities, typically in the duration range of 0.3–5 s. The main features of sound-motion objects are that they are multimodal, meaning that they include sensations of both sound and sound-producing body motion, and that they are conceived and perceived holistically as strongly coherent and stable units and hence may be called *objects* in musical performance.

Besides enhancing our understanding of the multimodal nature of music, such sound-motion objects are privileged both in the sound domain and in the human motion domain, by making present holistic features not found at shorter timescales, nor easy to focus on at longer timescales, for instance: An ornament object does not exist at timescales smaller than the duration of the entire ornament, and at larger timescales, the ornament will just be one element in a larger context, not enabling a focus on the features of that particular ornament. And in view of body motion constraints, there is, as we shall see, converging evidence that the typical duration of sound-motion objects may be optimal for both control and effort in sound-producing body motion.

The idea of sound-motion objects stems from our research on music-related body motion (see Godøy et al., 2016 for an overview), but is derived from Pierre Schaeffer’s theory of *sound objects* (Schaeffer, 1998, 2012, 2017; Chion, 2009; Godøy, 2021). The main idea of Schaeffer’s sound objects is that they have a limited duration and convey salient features linked with their overall dynamic, timbral, and pitch-related shapes. In Schaeffer’s sound object theory, these features may be further differentiated by a top-down and increasingly detailed classification scheme, however, always based on the subjective perceptions of these features as shapes (Godøy, 2021). The sound object focus of Schaeffer, although originating in connection with electroacoustic music of the 1940s and 1950s (Schaeffer, 2012), is very general and applicable to most kinds of music, regardless origin and genre, provided the point of departure for any inquiry is the subjectively perceived dynamic, timbral, textural, and pitch-related shapes (cf. a number of audio examples from different kinds of music in Schaeffer, 1998).

In addition, our own and other work of the last couple of decades on music-related body motion suggest that there are strong links between perceptual images of musical sound and sensations of body motion, be that as detailed images of *sound-producing motion* of performers, or be that as so-called *sound-accompanying motion* by listeners with more generic energy envelope resemblances between sound and other kinds of body motion, such as in walking, dancing, or gesticulating (Godøy and Leman, 2010; Maes et al., 2014). Actually, it may often be difficult to tease apart what are sound sensations and what are body motion sensations in music perception and/or musical imagery. For instance, in the case of ferocious drumming, what in our perception is due to the energy of the drum sound, and what is due to the sensation of energetic mallets, hands, arms, shoulders, torso, etc., motion of the performer that we see or just imagine? Or is our perception of the drumming made up of some combination of all these sensations?

From the perspective of the so-called *motor theory of perception* (Godøy, 2003; Galantucci et al., 2006), the answer is that we tend to perceive sound, albeit variably so, in terms of some mental images of how we believe the sound has been produced. To find out more about this, we previously made studies of so-called *air instrument performance* (Godøy et al., 2006) and of so-called *sound-tracing* (Nymoen et al., 2013). It seemed that listeners, with different levels of musical expertise, all tended to render some feature of musical sound by body motion; however, expert musicians tended to render more details of assumed sound-producing body motion.

In line with the motor theory claims of close connections between sound and assumed sound-producing body motion, the concept of *constraints* should be understood here not only in a limiting sense of possible vs. impossible, or easy vs. difficult, in performance, but should also be understood in a positive sense of providing the basic conditions for music performance. This means that various constraints may become an integral part of musical creativity and expression, even to the extent that we as listeners may actually expect the music to be in line with familiar constraints.

One outcome of this approach will be constraint-based shifts between muscle contractions and muscle relaxation and associated parsing of performers’ sound-producing body motion into chunks, i.e., into what we may call motion objects. In a striking parallel to sound objects, some human movement researchers have suggested a similar object perspective in the form of so-called *motor gestalts* (Klapp and Jagacinski, 2011). Motor gestalts are preprogrammed chunks of body motion that are triggered and carried out as holistic entities, thus optimizing motor control. In a similar vein, other research on human motor control has suggested that there is an intermittent, or discontinuous, element in body motion, meaning that there is a point-by-point triggering of motion based on a piecewise anticipatory planning of the motion to come (Loram et al., 2011). The combination of sound objects with intermittently triggered motor gestalts in body motion is then the basis for coining the term *sound-motion objects* in our research (Godøy et al., 2016).

The use of the word *object* in our present context is based on the observation that although both sound and motion are ephemeral and temporally distributed phenomena, they seem to leave some more solid traces in our minds, as well as to be conceived and perceived holistically at discontinuous points in time. This is the core meaning of “object” here, namely *a more or less solid mental image of a delimited fragment of unfolding sound and motion*. Crucially, this use of the word “object” does not signify something that is opposed to “subjective” sensations, but on the contrary takes individual-subjective perceptual images of sound and motion as integral to musical experience; however, there may in many cases be inter-subjective agreements about the experience of various features, as was indeed the point of departure for Schaeffer’s sound object theory. The term “object” here is then a collective term for sound and motion features as *shapes*, and the corresponding research method here is that of exploring shapes associated with sound-motion objects as well as the possible *correlations* between subjective object images and recorded sound and motion data (such correlation mapping is also a core element in Schaeffer’s theory, see Godøy (2021) on this). The epistemological approach here may be regarded as “object-focused” (the term “object-oriented” already taken and used in programming languages), in the sense that object images of sound and motion are the core components of our theory.

This object-focused approach touches on long-standing enigmatic relationships between notions of continuity and discontinuity in both philosophy and psychology, and which the concept of sound-motion objects seeks to address by what can be called the *intermittency hypothesis*. This hypothesis suggests that at the timescale of sound-motion objects (as mentioned, in the 0.3–5 s range), we may find compressed overview images of both sound and motion. This hypothesis also suggests that such “all-at-once” or “instantaneous” images of any segment of sound and motion can work both retrospectively (as recollections) and prospectively (as anticipatory control) and, hence, be temporally bidirectional.

The main aim of this paper is then to present the theoretical basis for constraint-based sound-motion objects in music performance, including the intermittency hypothesis as an attempt to work around some of these constraints. Given the explorative nature of this main aim, the present paper will be a so-called *hypothesis and theory paper*, but with some illustrative examples from our work on sound-producing motion.

For a start, it could be useful in the next two subsections of the present introductory main section to consider issues of *timescales* as well as of *constraints* in music performance. These subsections will be followed by a main section focused on *sound-producing motion*, containing four subsections concerned, respectively, with *motion features* and phenomena essential for the fusion of sound and motion into coherent entities, so-called *phase transition* and *coarticulation,* as well as the phenomenon of *idioms* of sound-producing motion, i.e., what may be considered particularly successful sound-motion objects. Then follows a main section on *control theories*, containing three subsections concerned with *motor control*, *motor gestalts* and the so-called *posture-based theory*, a theory suggesting that motion is centered on salient postures at selected moments in time. All this will be seen to converge in the main section on *musical intermittency*, containing subsections on *the intermittency hypothesis*, the *triggering* of motion chunks, ending with the concept of *sound-motion objects*. Finally, there will be a discussion section on the various advantages and challenges of the sound-motion objects perspective on music performance.

### Timescales

The concept of sound-motion objects depends crucially on duration criteria. To see why this is the case, it could be useful to have an overview of the different timescales for sound and body motion features in music performance. These timescales extend from those in the sub-millisecond range to those in the range of several seconds, minutes, and beyond, here grouped into three main categories (Godøy, 2006):

- *Micro*, denoting the below ≈ 300 ms event timescale and encompassing stationary sound features such as pitch, timbre, dynamics, as well as some fast transients and fluctuations, including salient timbral-textural features in the duration region of around 250 ms (Gjerdingen and Perrott, 2008).
- *Meso*, typically the timescale of sound-motion objects in the mentioned duration range of 0.3–5 s. This is the timescale of the most salient sound object features in Schaeffer’s theory as well as in more traditional Western music theory, such as motives, ornaments, contours, and modality conveying features of style and affect, as well as sense of corresponding body motion. There may very well be a general predisposition for the meso timescale as suggested by Ernst Pöppel in that the approximately 3-s time window is optimal for human perception and cognition in several domains (Pöppel, 1997). The meso timescale also corresponds to so-called *short-term memory* where basic processing and feature extraction are assumed to go on (Snyder, 2000).
- *Macro*, denoting the longer than meso timescale, with durations in the range of up to minutes and even hours. However, the efficacy of these large-scale forms as proclaimed by mainstream Western music theory, as well as by Schenkerian analysis, could be questioned (Eitan and Granot, 2008). There have been debates between proponents of more meso timescale views and more macro timescale views, for instance with Jerrold Levinson’s “concatenationism” vs. Peter Kivy’s “architectonicism” (Levinson, 2015). In our present context of constraint-based sound-motion objects, the main point is to see the convergence of several salient sound and motion features in the meso timescale of sound-motion objects, hence that there is an affinity here with Levinson’s ideas, yet also recognizing that there may always be some larger context for these objects, and that the focus in our perception may vary between different timescales.

For sound-motion objects, we may also have different nested timescales, extending from that of the entire sound-motion object down to that of its internal spectral content, but where the meso timescale is privileged in manifesting the convergence of several different perceptual-cognitive features. Interestingly, the initial reason for Schaeffer’s focus on meso timescale sound objects was the following two experiences (Chion, 2009; Schaeffer, 2017):

- The experience of countless repeated listening to looped fragments of sound in the early days of electroacoustic music, the so-called *closed groove* experience, emphasizing the crucial importance of the overall dynamic, timbral, and pitch-related shapes of the sound objects.
- The experience of the so-called *cut bell*, of how the different parts of any unfolding sound contribute to the total perceptual image of the sound, in particular how the dynamic shapes of the attack segments color the perceptual image of the subsequent stationary segments of sounds.

These two experiences demonstrated that sound features are temporally distributed, and hence, that a sound object should take the entire sound fragment into account.

For these features to become perceptually manifest, Schaeffer added the constraint of the so-called *suitable object* which included some duration and content criteria. Durations should be sufficient to encompass salient sequentially unfolding events, e.g., attack and sustain segments, yet not too long, nor too diverse, or too static, and the examples given in (Schaeffer, 1998) are mostly in the mentioned 0.3. to 5 s range, sometimes shorter and in exceptional cases longer, i.e., up to 30 s. The too short ones can become acceptable with pauses between the objects, so that a pause actually becomes part of an object. Furthermore, an important feature of sound objects is what Schaeffer called *facture*, essentially denoting the energy envelope of the sound object and making a link with body motion, and hence, also making a link also with the timescales of body motion (Godøy, 2006).

Furthermore, timescales concern not only duration issues, but also issues of continuity vs. discontinuity in perception and cognition. This was much discussed in philosophy and psychology toward the end of the nineteenth and beginning of the twentieth century. Edmund Husserl argued that perception and cognition proceed in a discontinuous manner by a series of so-called now-points, points in time for a cumulative and prospective overview of a segment of sensory unfolding, and that without such intermittent stepping out of the sensations stream, we would not be able to extract any meaning from our experiences (Husserl, 1991; Godøy, 2010).

Husserl’s model of a point-by-point overview image of past and future events is relevant for a number of present issues in perception and cognition, *cf.* Schaeffer’s view of sound object perception needing to take the entire fragment into account. And in the case of motor control, Husserl’s model resembles a discontinuous, point-by-point scheme of anticipatory motor control as manifest in the ideas of motor gestalts and intermittent control. This means that timescale issues are closely linked with core issues of motor control, and as we shall see, in particular with the intermittency hypothesis, for instance, the idea of “segmented control” (Sakaguchi et al., 2015) seems to resemble this “now-point” view of cognition.

At the object timescale, motion control and motion effort have been referred in some of the intermittency literature as “serial ballistic,” “open loop,” or “feedforward,” terms essentially expressing the view that at this timescale, there is a discontinuity of control and effort, yet seen from the outside, the resultant sound and motion may appear to be continuous.

### Constraints

Taking instrument and body motion constraints of music performance into account means taking a concrete, that is, a non-abstract, approach to music cognition. Furthermore, this means regarding symbols of Western music notation as a sparse script for concrete sound-producing motion to be manifested on concrete instruments, necessitating a transformation and an adaptation to a set of combined instrument-motion constraints.

As for instrument constraints, they include both acoustic and ergonomic features. The various modes of excitation, such as hitting, plucking, stroking, and blowing and corresponding modes of resonance, form the basis for sound-motion objects. Singular instrument sound objects may in turn have multiple internal features, e.g., the grainy quality of bowed deep double bass tone, or the buzzing sound of a snare drum.

On top of the instrument (and room) constraints, body motion constraints are shaping the sound-motion objects, first of all by the obvious fact that all body motion takes time, i.e., that there is no instantaneous displacement of the sound-producing effectors (fingers, hands, arms, feet, vocal apparatus). This means that sound events are embedded in motion events, and that there may be fusion of motion events by the phenomena of so-called *phase transition* and *coarticulation*:

- Phase transition in motion contexts denotes a categorical change in motion mode based on incremental changes in speed or rate of motion (Haken et al., 1985), for instance, between protracted singular strokes and fast tremolo motion in bowing, with emergent constraint-based changes in the motion amplitude of the bow.
- Since all body motion takes time, there will always be a contextual smearing in the form of coarticulation, meaning the contextual fusion of otherwise separate sound and motion events into new and longer sound-motion objects (Godøy, 2014).

There will be more details on these fusion phenomena later, and for now, we should not forget some other constraints of sound-producing motion, such as limitations of speed and endurance, need for rests, and need for motion strategies to avoid strain injury (Altenmüller et al., 2006), all contributing to the shaping of sound-motion objects. In terms of optimization of sound-producing body motion, we may also note:

- There seems to be a tendency toward minimization of energy expenditure in expert musicians (Winges et al., 2013).
- Fluency is a hallmark of experts’ minimization of effort as opposed to non-experts’ clumsiness (Gonzalez-Sanchez et al., 2019).
- Concrete, non-abstract, and logistic-ergonomic motion is often needed, e.g., in drum set performance (Godøy et al., 2017).
- Patterns of sound-producing motion that are particularly successful in generating good-sounding results with minimal effort, known as *idioms*, are important in the context of sound-motion objects because they testify to the extensive fusion of sound and motion optimization.
- On the other hand, implementing motion constraints may drastically change features of the output sound (Rozé et al., 2020).

It may sometimes be difficult to distinguish what are basically cognitive control constraints from what are more biomechanical constraints; however, there seems to be agreement that there is a control constraint with the so-called *psychological refractory period* (PRP). The PRP is believed to impose a limitation of around 0.5 s for initiating new motion in the course of any currently ongoing motion (Klapp and Jagacinski, 2011) and has the following consequences:

- A workaround solution to the PRP constraint may be anticipatory motor control in the form of the mentioned motor gestalts, meaning that an entire motion chunk may be carried out without the need for attention to details. This means that with PRP making continuous feedback control impossible, PRP leads to intermittent control, and hence, intermittency is constraint-based (Loram et al., 2014).
- Recognizing that all control processes in human motion take time because of inherent speed limitations of the neurocognitive apparatus, there have been long-lasting discussions of so-called *open loop* (no continuous feedback) vs. *closed loop* (continuous feedback) in human motor control; however, there may now be some kind of half-way agreement, cf. (Hanneton et al., 1997; Desmurget and Grafton, 2003), as also suggested by the intermittency hypothesis.
- Differentiating timescales could be a solution to *feedback* vs. *feedforward* disagreements in the sense that they can coexist as interleaved phenomena, i.e., assuming feedforward control being the case for rapid and continuous motion; however, it can alternate with feedback control at intermittent intervals (and with subsequent error correction), as suggested by the principle of “observe continuously, act intermittently” in intermittent control (Loram et al., 2011, p. 317).

In sum, constraints concern both motion and the control of motion; hence, the next sections will be about basic motion features, first from the more biomechanical and bottom-up constraints point of view, followed by the more top-down control point of view of constraints, and after that, there will be a focus on what are basically optimization elements, including the intermittency hypothesis.

## Sound-Producing Motion

### Motion Features

Music-related body motion is often differentiated into the main categories of sound-producing motion, e.g., such as hitting, stroking, kicking, bowing, blowing, and sound-accompanying motion, e.g., dancing, walking, gesticulating (Godøy and Leman, 2010). Although the boundaries between these two main categories may sometimes be blurred, such as in cases of theatrical motion by musicians, the main feature of sound-producing motion is that of contributing to the generation of musical sound. This will first of all include what we call *excitatory motion*, i.e., motion that transfers energy from the body to an instrument, but it will also include so-called *modulatory motion*, such as for changing pitch or timbre, as well as *ancillary motion*, for instance, for optimizing postures and help avoid strain injury as well as help in shaping musical expression (Cadoz and Wanderley, 2000).

Furthermore, there are a number of attributes of sound-producing motion such as trajectory shape, amplitude, velocity, acceleration, and periodicity that may all variably contribute to the features of the output sound, so much so that sound-producing motion and output sound may fuse into our sound-motion objects. With available technologies and analytic tools, it is possible to zoom in on details of motion, for instance, into the finger acceleration rates that pianists use for different types of articulation (Palmer, 2013).

In rather broad terms, we can differentiate sound-producing motion into what we call *typological categories* as was suggested by Schaeffer for sound objects (Schaeffer, 2017). By way of the mentioned *facture*, this typology reflects distinct biomechanical and motor control features with its three main categories:

- *Sustained*, denoting a continuous, protracted sound corresponding to a continuous transfer of energy from the body to the instrument such as in bowing or blowing.
- *Impulsive*, meaning a short and abrupt sound, such as produced by hitting or plucking.
- *Iterative*, denoting a rapidly repeated sound, such as in a tremolo or trill, produced by a corresponding rapid shaking or rotating motion.

**Figure 1:**
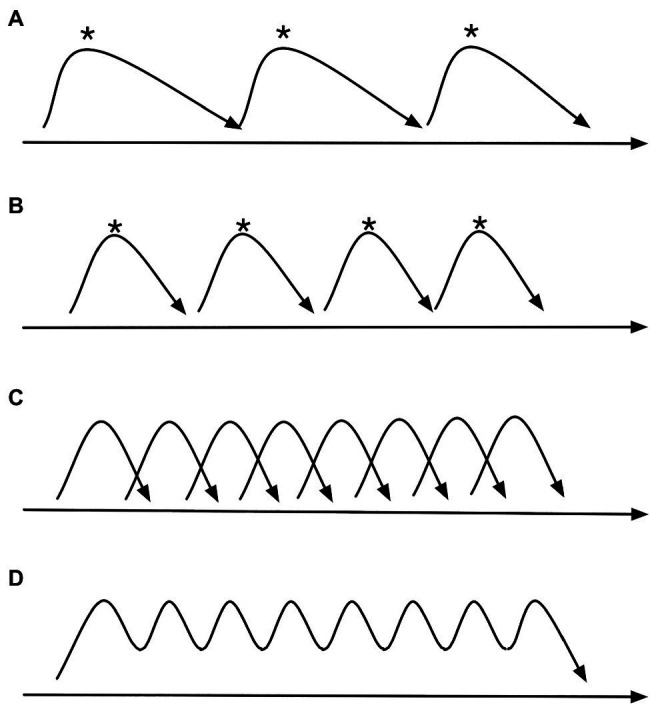
A schematic image of emergent phase transition. If a series of separate motion events, each with a goal posture marked with an asterisk, lined up along a timeline from left to right such as in **(A)**, are moved closer together in time like in **(B)**, and even closer still, the events will fuse together like in **(C)**, becoming more like an iterative pattern where the originally separate events have become transformed into a rapid wavy motion like in **(D)**.

**Figure 2:**
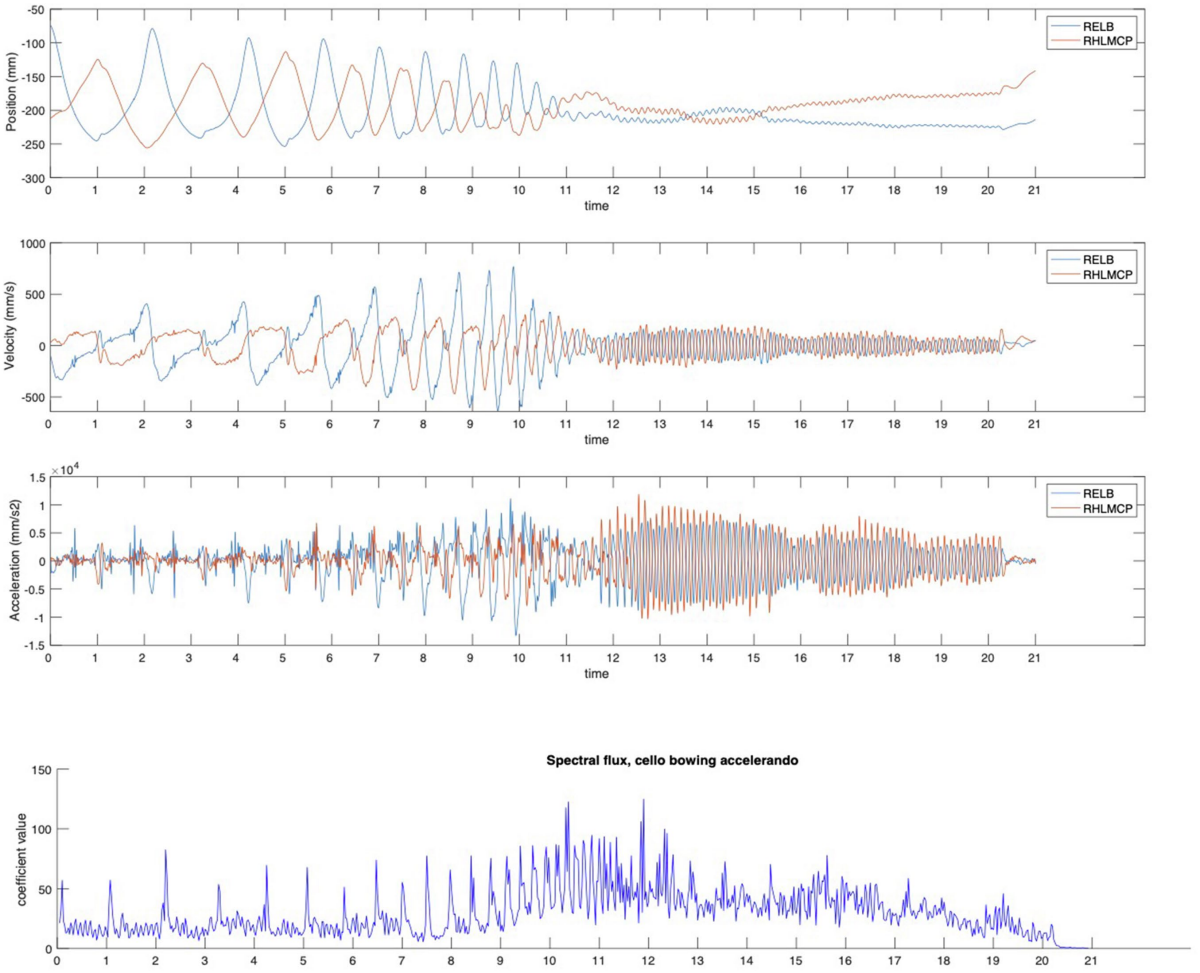
Phase transition in bowing. The figure displays the markers placed on the elbow and wrist (the right hand metacarpophalangeal articulation, next to the left hand little finger) tracing the back-and-forth motion of cello bowing, accelerating from slow to very fast (as fast as in a tremolo), is displaying phase transition of the motion patterns due to the constraints of the high speed, i.e., a change to smaller amplitude motion because large amplitude motion would be impossible at high speeds, eventually ending up in just a shaking of the wrist at the highest speed. Also, note the changes in the spectral flux (rate of change of spectral features) due to an increase in the noise components in turn due to the increase in transients because of the increase in the rate of bow direction changes. The motion trajectories of the two markers were recorded using a Qualisys motion capture system at a frame rate of 300 fps and analyzed with the MoCap Toolbox software (Burger and Toiviainen, 2013), and the spectral flux of the sound of the F#4 was calculated using the MIRtoolbox software (Lartillot and Toiviainen, 2007).

There are categorical thresholds between these main types, and we may move from one to another by the earlier mentioned phase transition. For instance, a sustained sound and motion may turn into an impulsive sound and motion if shortened, and a series of impulsive sounds and motion may turn into an iterative sound and motion with increasing rate. Examples of such phase transitions can be seen in Figures 1 and 2.

The main purpose of this motion typology is to point out the close links between musical sound features and motion features. This is in particular relevant in view of the emergence of coherent sound-motion objects due to the sound-producing motion with intermittent control and energy input.

### Phase Transition

The expression *phase transition* has been used to signify qualitative changes in physical substances on the basis of continuous or incremental change in some underlying parameter, e.g., the change from ice to water to steam on the basis of changes in temperature, but has also come to signify qualitative change in other phenomena such as mode of motion, for instance, with changes from walk to trot to gallop on the basis of the animal’s speed of motion. Furthermore, phase transition has also been used to signify emergence of various cognitive phenomena (Haken et al., 1985; Spivey et al., 2009).

Taking a wide view of phase transition, we could include several perceptual phenomena ranging from low-level features such as the flicker-fusion threshold in vision and the similar threshold in auditory perception between impulses and pitch, to higher level categorical phenomena such as the identification of timbre, interval categories, various rhythmical patterns, and melodic contours with the common element of categorical threshold crossings resulting from continuous or incremental change of some underlying parameter.

Given the constraints of human body motion, we may assume that phase transition can be found in music performance, in particular with repetitive motion such as in bowing (Rasamimanana et al., 2007). We can see two illustrations of this in the present paper. In Figure 1, there is a schematic depiction of phase transition starting from a series of separate events, and if these events are shortened and moved closer, e.g., if there is a tempo increase in the music, we see that the events will overlap and the series of distinct events will be transformed into a stream of undulating motion. In Figure 2, we see a similar case of phase transition, but here with accelerated bowing on a cello. In the initial slow tempo, we see the long bowing motion of both the elbow and the wrist, but with the acceleration, the bowing motions become shorter until the bowing reaches the speed of a fast tremolo and where bowing is reduced to just a small-amplitude wrist shaking motion. Interestingly, this phase transition of bowing motion has also resulted in significant timbral changes, with the sound becoming more noise-dominated as can be seen from the graph of spectral flux, i.e., an indication of degree of spectral change along the temporal axis.

Phase transition is about emergent (and forced) fusion of initially separate events (both sonic and sound-producing), but may just as well be about the fission of coherent events into separate events. For instance, we may also come across phase transition from fast to slow, sometimes also showing the difficulties of moving slowly (Park et al., 2017).

### Coarticulation

The term *coarticulation* signifies the fusion of otherwise distinct motion events into larger and more coherent motion events. Coarticulation is found in several domains of human motion, first of all in linguistics (Hardcastle and Hewlett, 1999), but also in other areas such as typing, hand writing, sign language, and various everyday human activities, see (Rosenbaum, 2009) and (Grafton and Hamilton, 2007) for general presentations, as well as (Sosnik et al., 2004) for emergent coarticulation by practice and optimization. But we also have some publications on music-related coarticulation such as the following:

- In piano playing, with fingers moving to optimal positions before hitting keys (Engel et al., 1997).
- Coarticulation and so-called *muscle synergy*, i.e., cooperation between muscles groups, in piano performance (Winges et al., 2013).
- In string playing, with left hand fingers in place in position well before playing of tones (Wiesendanger et al., 2006) and in the contextual smearing of bowing movements (Rasamimanana and Bevilacqua, 2008).
- In drumming, where a drummer in some cases may start to prepare an accented stoke well in advance (Dahl, 2006).
- For some examples of our own work with motion capture data of piano and marimba performance (see Godøy et al., 2010; Godøy, 2014).

Coarticulation may involve different elements, and all of them may contribute to the fusion of otherwise separate elements in music performance as in the following (Godøy, 2014):

- *Temporal coarticulation*: otherwise singular events become embedded in a context.
- *Spatial coarticulation*: motion in one effector (e.g., hand) recruits motion in other effectors (e.g., arm, shoulder, torso).
- *Spillover effects*: past events influence present events, i.e., position and shape of effectors are determined by recent motion.
- *Anticipatory effects*: future events influence present events, i.e., position and shape of effectors are determined by preparation for future motion.

**Figure 3:**
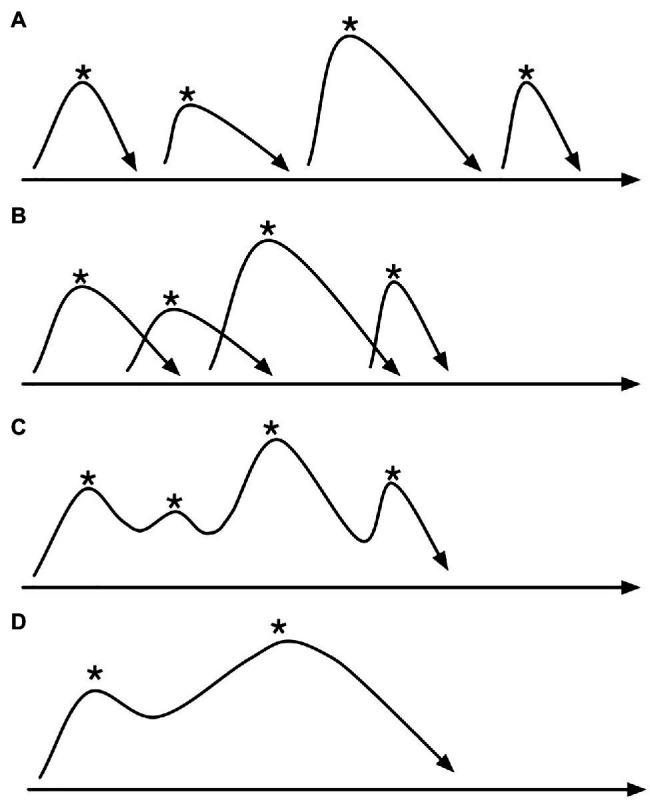
A schematic image of emergent coarticulation, starting with four separate events along a time line from left to right such as in **(A)**, each event centered on a goal posture marked with an asterisk, then moved closer together in time in **(B)**, with the consequence that the partially overlapping trajectories become more like a continuous trajectory in **(C)**, and resulting in an even more smoothed trajectory in **(D)**, thus illustrating the event fusion phenomenon of coarticulation.

**Figure 4:**
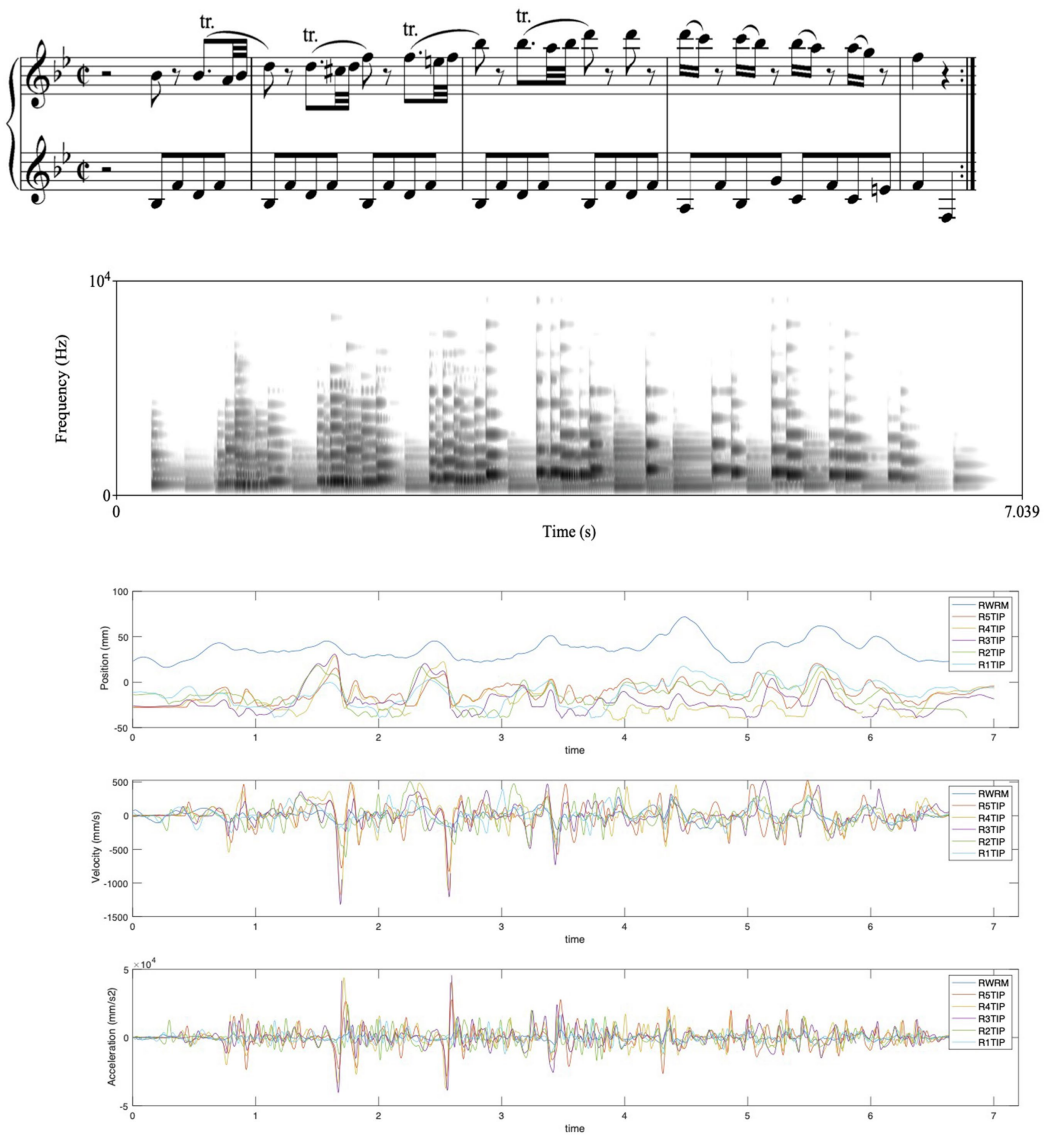
Coarticulation in a piano performance, here of the first four measures of Mozart’s Variations KV500, showing the notation, a spectrogram of a performance of these measures, and the vertical motion data (the “z” dimension) of the five fingers and the wrist of the right hand from this performance, including the positions, velocity, and acceleration of the fingers/wrist. The burst of finger motion for each of the trills is most clearly seen in the velocity and acceleration plotting. The motion trajectories of the five fingertips and the wrist of the right hand markers were recorded using a Qualisys motion capture system at a frame rate of 250 fps and analyzed with the MoCap Toolbox software (Burger and Toiviainen, 2013), and the spectrogram of the sound was made with the Praat software (Boersma, 2001). The notation (score) is added for reference purposes and aligns only approximately with the recorded data.

Coarticulation seems to be quite common in music performance, and here just two illustrations of this. In Figure 3, there is a schematic depiction of how we may think of coarticulation as emerging from a context of sound-producing events, initially consisting of a series of separate events, but when these events are moved closer together, there will be a contextual spillover effect that is the hallmark of coarticulation, successively blurring the boundaries between the events until the original events fuse to become more like a continuous motion trajectory. In Figure 4, we can see how a series of trills result in a similar smearing of finger motion because of the need for rapid motion in playing the trills.

As for the more detailed workings of coarticulation, the score-like depictions of muscle synergies of (d’Avella and Lacquaniti, 2013), showing how different muscles participate at different moments in time, are highly relevant for how we believe coarticulation for more complex sound-producing body motion may be implemented and is something to be explored further, cf. attempts in this direction in (Winges et al., 2013).

As an ubiquitous phenomenon in music, coarticulation provides a testimony of constraint-based contextual smearing of events. Coarticulation means that performers are constantly preparing for the upcoming sound-motion events, unless there is a pause (with no motion) ahead. This contextual smearing of events due to coarticulation is in a sense what makes music (and spoken language) sound “natural,” and recuperating the “original” events behind the smeared manifestations of coarticulation can be very challenging. We have seen some interesting work in this direction (Bevilacqua et al., 2016), and furthermore, this recuperation could also resemble recovering underlying intermittency components from emergent continuous sound and motion.

### Idioms

The term *idiom* in connection with music performance denotes particularly successful sound-motion objects in the sense of having maximally aesthetically pleasing audible results with minimal effort, in view of both biomechanics and control, of sound-producing motion. As any quality textbook on orchestration can tell us, idioms in music is a huge topic, yet it should be mentioned in our context of sound-motion objects because it is crucial for how musical expression can emerge from constraints, as well as for how composers’ and arrangers’ considerations of such constraints may influence the music they produce.

Consider, e.g., the case of tremolo by back-and-forth bow motion for repeated tones as easy in string instrument performance (cf. the cello bowing phase transition example above), but difficult in a keyboard performance, whereas a tremolo by an octave rolling motion on keyboards would be easy. Conversely, a rapid octave back and forth motion on a string instrument would be difficult and hence is something that would better be rendered as single tone repetitions on a bowed string instrument (cf. Godøy, 2018 for examples of this). Also, same tone repetitions can be done with relative ease on some other instruments, e.g., brass instruments, but not so easily on instruments that have the mouthpiece inside the mouth, e.g., woodwind instruments. Furthermore, so-called *flatterzunge* is easy on some and difficult on other wind instruments. Related, vocally, making an alternating sound such as *ti-ku-ti-ku* is easier than a non-alternating sound such as *ti-ti-ti-ti*.

These examples concern biomechanical issues, but there are also types of sound-producing motion that are challenging more on the control cognitive side, e.g., some tones, typically in the extreme registers, may be difficult or risky to produce, whereas others are failsafe, such as open strings tones and lower natural harmonic tones on string instruments. Also, there are cognitive control issues, i.e., mental load because of too many and/or rapidly changing patterns, e.g., unconventional bowing patterns exemplified in (Rosenbaum, 2009, p. 124), or rhythmically extremely complicated patterns that place a heavy cognitive load on the musicians. Additionally, there are cases of active cultivation of idioms for the sake of maximal output with minimal effort (both biomechanical and cognitive), such as in the mature works of Rimsky-Korsakov where practically every single part in the orchestral texture has been designed in view of optimal idiom-compliance.

In Western music culture, the idea of going from score to performance by way of so-called *interpretation* could here be understood as an optimalization of sound-producing motion by way of the mentioned principles of phase transition and coarticulation, i.e., minimizing effort by constraint-compliance. Interestingly (Furuya and Yokota, 2018) demonstrated optimization of effort by rhythmical variation, denoted “neuromuscular adaptation,” to find the most favorable mode of motion, and the removal of unnecessary motion components by expert musicians can increase the sense of fluency in performance (Gonzalez-Sanchez et al., 2019).

## Control Theories

### Motor Control

One core idea of sound-motion objects is that of a within-object coherence, both of the sound and of the corresponding body motion. To broaden our understanding of this coherence, it could be useful to have a look at coherence-enhancing elements of motor control in general, followed by the more specific theory of *motor gestalts*, as well as a theory of so-called *goal postures*, which are postures at particularly salient moments in human motion. Motor gestalts and goal postures are both elements of human motion that lead to the *intermittency hypothesis*, i.e., the hypothesis of the discontinuous, point-by-point emergence of sound-motion objects.

From an overview of human motor control (Rosenbaum, 2009), and more specifically of music-related motor control (Altenmüller et al., 2006), there seems to be a basic agreement that skilled behavior necessitates extensive practice as well as using perceptual corrective feedback during performance; however, there seem to be divergent opinions about the rate and speed of such corrective feedback. Also, there is variable concern about the biomechanical constraints involved in skilled behavior, i.e., there seems to be not always a focus on the physical effort required, cf. the motion constraints mentioned above.

What there seems to be agreement on though is that there are inherent limitations on the speed of the human motor control system, particularly concerning reaction times, hence the need for some kind of anticipatory element to work around such limitations. Yet there seems to be disagreement about the duration of such reaction times as well as about the extent of the corresponding anticipatory pre-programing in human motion. Some researchers have seen this as a debate lasting more than a century (Elliott et al., 2001), dating back to Woodworth’s seminal 1899 paper on the so-called *initial impulse* in motion, designating a first and typically coarse motion trajectory that needs to be corrected when homing in on a target.

A related issue is the need for simplification of human motor control by way of hierarchies with the idea of a top-down chain of command starting with the overall executive goal being passed on to various lower-level motor control components, resulting in coordinated sequences of muscle contractions and relaxations that finally make the wished for motion to happen (Grafton and Hamilton, 2007). Following the pioneering writings on goal-directed motion by Nikolai Bernstein, it is suggested that motion is controlled in a hierarchical manner which also includes the integration of different motor units into a single functional unit, manifest as a coarticulation cooperation between the motor units.

Another recurring and relevant concept is that of *motor programs* (Summers and Anson, 2009), denoting the principle of some kind of mental script for a motion sequence, which also concerns the degree of pre-planning and degree and rate of corrective feedback in the course of implementing the motion sequence (Desmurget and Grafton, 2003). The topic of corrective feedback is related to general *control theory*, a topical area situated at the intersection of engineering and human movement science. Control theory spans from rather simple regulatory systems such as thermostats to highly complex and human-like control systems and includes key concepts such as *open loop*, *closed loop*, *feedforward*, and *feedback*, now also elements of predictive and intermittent human motor control. Most of these concepts are relevant for motor control in music performance; however, we need as always to consider the timescale in question: Some features of music happen so fast that closed loop feedback will be impossible, making musicians rely on preprogramming and open loop, feedforward control, and hence, on motor gestalts in combination with intermittent corrective feedback.

### Motor Gestalts

In (Klapp and Jagacinski, 2011), we find a strong claim for pre-programing in their notion of *motor gestalts*. The gist of their reasoning is that motion control shares several features with gestalt perception, first of all in working by holistic entities, something that is regarded as the quintessential feature of perceptual gestalts.

The extensive and long-lasting relationship between gestalt theory and music attests to the affinity of basic gestalt principles and music perception. It should be remembered that gestalt theory actually had its beginnings in music perception with the work of Ehrenfels, Stumpf, and others (Smith, 1988), and ensuing gestalt-related publications attest to a continued relationship (e.g., Tenny and Polansky, 1980; Bregman, 1990; Leman, 1997). Gestalt theory was also influential in the development of Schaeffer’s sound object theory (Schaeffer, 2017), and although there have been a number of divergent opinions on details of gestalt theory, the main idea is clear, namely the “all-at-once” presence of fragments of sensory experience as objects, underlining our organisms penchant for organizing also sound and motion as objects. The idea of motor gestalts is based both on the general features of gestalt and on the need for an efficient and reliable way to control body motion given the limitations of the human motor control system. The most serious challenge for the human motor control system is the mentioned PRP; hence, a reliable possibility of pre-programming would be advantageous.

The core of gestalt theory is the following: “Perhaps the most fundamental aspect of the gestalt tradition is the notion of a gestalt as a *holistic pattern* that is ‘more than the sum of its parts.’ Thus, it is a unit rather than isolated components.” (Klapp and Jagacinski, 2011 p. 444). A crucial feature of gestalt is then that of something solid, and in the case of motor gestalt, something that is ready to be instantiated very quickly. Measuring so-called reaction time, or RT for short, has been used for probing the degree of preprogramming. So-called *Simple RT* designated the triggering of a ready response, whereas *choice RT* designated the need to assess and choose the most suitable response. In our context, simple RT seems to be the most relevant as an indicator of ready-made motor gestalts. If the RT increases, it is assumed to be due to having to choose between different responses.

Motor gestalts are seen to have the following four principles in action, as summarized in (Klapp and Jagacinski, 2011):

1. “A brief chunk or motor gestalt is a holistic pattern.” (*Ibid*, p. 458). It has previously been suggested that alternatively, the word *chunk* could be used to designate a motor gestalt provided that “it (a) is processed as a unit and not as separable components and (b) functions to enable a coordinated action.” (*Ibid*, p. 444).
2. “Abstract coding permits perceptual constancy; an abstract action code is not tied to specific effectors, thereby permitting constancy in motor control.” (*Ibid*, p. 458). Such a flexibility of effectors seems similar to what is sometimes called *motor equivalence*, i.e., that different effectors can make similar motion patterns, as well as do so at different scales, e.g., signature on a small piece of paper or on a large blackboard.
3. “Motor gestalts are mutually exclusive; only one gestalt can be programmed at any moment.” (*Ibid*, p. 458). This is crucial for rhythmical patterns in that a series of sound onset motions cannot at the same time belong to two different metrical organizations, e.g., 6/8 and 3/4, by the principle of so-called *exclusive allocation*. In another publication, there is the following passage concerning polyrhythm: “The limitation to only one motor Gestalt may be analogous to limits that arise with visual patterns such as the Necker cube. That figure can be perceived in only one of its configurations at any given instant. In either configuration, however, all of the lines of the cube are perceived simultaneously as one pattern. Thus, the Gestalt is not restricted in terms of the number of lines that can be perceived. Instead, the limit is that only one organization can be activated. Similarly, the limit in concurrent motor actions is assumed not to lie in the number of muscles that can be controlled, but, instead, the limit is that only one action pattern can be active.” (Klapp et al., 1998, p. 318). In other words, even rather complex motion patterns may be conceived and perceived as single gestalts in motor control.
4. “The organization of an action sequence can be either integrated or streamed.” (Klapp and Jagacinski, 2011, p. 458). This is related to so-called *streaming* in auditory scene analysis (Bregman, 1990), meaning that a sequence of sound events may either be seen as belonging to several parallel streams, or as belonging to one single stream, as suggested for a polyrhythmic pattern transformed from two streams to one single integrated entity, e.g., the 3 against 4 streams transformed to a single punctuated rhythmical pattern. Interestingly, the suggestion that “Speed of action can influence organization” (*Ibid*) seems similar to categorical shifts due to the above-mentioned phase transition.

The fundamental meaning of motor gestalts in music is then that it is conceived and perceived as a holistic entity, and as having a coherent motion trajectory from start to end, hence, also contributing to the coherence of sound-motion objects in music performance.

### Posture-Based Theory

One way to look at sound-motion objects is that they are hierarchically organized around goals. This goes back to the pioneering work of Bernstein with his idea of the complexities of human motion and the multiple degrees of freedom and associated redundancy (i.e., multiple solutions possible to any task) needing to be regulated by some kind of hierarchical control scheme. There are different models of hierarchies in motor control, but usually with some scheme for simplification and automatization of lower level control tasks. Such top-down control schemes have the advantage of optimizing the motion components that go into any sound-motion object, hence, also linked with coarticulation [see (Grafton and Hamilton, 2007) on this as well as an interesting discussion of Bernstein goal-directed behavior]. A similar focus on goals has been a leading idea in David Rosenbaum’s work on motor control, such as in the case of dance:

“Dance, for its appearance of being a *continuous* activity, is actually controlled, or is supposed to be controlled, by aiming for one target position after another. Insofar as this method is endorsed by dance coaches and proves useful for dancers, it probably reflects a deeper principle about the control of physical action. That deeper principle, according to the posture-based motion planning theory developed by my colleagues and me, is that a reference condition for goal postures is established for positioning movements before movements to those goal postures are planned.” (Rosenbaum, 2010, p. 44)

Furthermore, according to Rosenbaum: “Dance, for its appearance of being a *continuous* activity, is actually controlled, or is supposed to be controlled, by aiming for one target position after another.” (*Ibid*, p. 44). Designated by the term *goal postures*, this is depicted as a general element of motor control in the work of Rosenbaum, also related to the phenomenon of *keyframes* in animation (Rosenbaum et al., 2007). The role of keyframes in animation is to establish salient moments in the narrative, with the aim of then making continuous motion between these keyframes, which given the keyframes becomes a much simpler task. Actually, some choreographers and dancers have been using a similar scheme in rehearsing new scripts called *marking,* denoting a sparse running through of dance sequences with basically just moving from posture to posture, not doing much motion between the postures, both in order to focus on the overall structure of any dance sequence, and to save energy during rehearsals (Kirsh, 2011; Warburton et al., 2013).

More recently, Rosenbaum has proposed this focus on postures as a general theory of motor control called *posture-based theory* (PBT), in particular associated with manual postures in body motion (Rosenbaum, 2017). Posture-based theory is then a theory of hierarchies in motor control, where motion is planned by way of the goal postures. It is interesting to see the similarity here with coarticulation in that there is a transition from one posture to another, i.e., that there is a temporal smearing effect in the motion trajectories. Furthermore, there is a link with intermittency with the temporally distinct goal postures, hence, there is a convergence here of intermittency, postures, motor gestalts, and coarticulation.

In sum, PBT is about the primordial importance of shape and position of effectors, and we can adapt this to music performance, primarily as postures of hands, but possibly also of other effectors such as arms, shoulders, torso, and feet, and not to forget the vocal apparatus. The position and posture shapes of effectors in relation to pitch space, as well as multidimensional timbre space, at salient moments in time, are that which (following the idea of PBT) could make the control of subsequent motion between postures easier. With both motor gestalts and PBT converging in intermittency, this may be included in what we call *shape cognition* (Godøy, 2019), in turn related to the general shape cognition of so-called *morphodynamical theory*, an extensive theory on the primordial role of shapes in perception and cognition (Thom, 1983; Petitot, 1990; Godøy, 1997).

## Musical Intermittency

### The Intermittency Hypothesis

The word *intermittent* has been defined as “not happening regularly or continuously; stopping and starting repeatedly or with periods in between” (Cambridge English Dictionary, 2021), hence as basically signifying discontinuity. Using the expression *musical intermittency* in this paper is then motivated by the need to have a general concept for discontinuity in musical experience.

In our context of music performance, such discontinuity may seem paradoxical if we think of music as a continuous stream of sound and motion sensations, cf. the mentioned similar paradox in dance (Rosenbaum, 2010). In our present perspective, it is actually this *coexistence of continuity and discontinuity* that is the crucial attribute of sound-motion objects, in that we may have discontinuity between the objects combined with subjective experiences of musical performances as a continuous stream.

Continuity vs. discontinuity may in our context be regarded as relative to timescale of observation, with the idea that intermittency, and hence discontinuity, is valid when we consider music as a series of sound-motion objects, and that we also have a within-object continuity, recall that this is typically in the 0.3–5 s duration range. Also, recall that the holistic perception of sound objects was one of the crucial features of Schaeffer’s theory, because the features of sound objects are spread throughout the sound object, and that the perception of such sequentially unfolding sound requires a cumulative, holistic perception, as demonstrated by the cut bell and closed groove experiences.

The relationships between discontinuity and continuity in music can been seen in relation to a more extensive debate in human motor control. Since the mentioned essay by Woodward from 1899 and ensuing debates (Elliott et al., 2001), a view of motion control as discontinuous emerged with the concept of intermittent control, initially proposed by Kenneth Craik in his posthumous publication (Craik, 1947) and in publications by his associate Margaret Vince (Vince, 1948). The term “intermittent ballistic” was used to denote control actions as being intermittent and having a character of ballistic motion, i.e., of an impulse followed by energy dissipation, as for instance when tapping a joystick. Similar ideas of intermittency in human motor control were presented by Navas and Stark (1968) and later on by Ronco (1999), and by several others in the ensuing decades (Gawthrop et al., 2011; Loram et al., 2011, 2014; Karniel, 2013; Sakaguchi, 2013; Sakaguchi et al., 2015). There are also intermittency-related ideas in other research, such as in research on hierarchical control in human movement (Grafton and Hamilton, 2007) and in research on muscle synergies (d'Avella and Lacquaniti, 2013). *Muscle synergies* here basically denote scripts of time-dependent contraction and relaxation of muscles ensembles, a muscular cooperation needed to produce the desired motion events, hence also related to coarticulation.

These different ideas can be seen to converge in what could be called the *intermittency hypothesis*, implying intermittency in control as well as in effort (or energy input), when applied to sound-motion chunks. The basic model for this hypothesis is that of an intermittent burst of effort followed by a prolonged phase of continuous motion, as suggested by the “serial ballistic” expression of Craik.

The reasons given for intermittency in the literature referred to above are first of all that an open loop control scheme with only intermittent feedback may be an efficient workaround for the slow and noise-prone motor control system, so that a series of intermittent motor control input points may be better able to handle the demands of performance than any attempt to have a continuous feedback, or a closed loop, control scheme. This is partly supported by behavioral evidence, but there are still substantial challenges of method in detecting these intermittent control points in time. For instance, when intermittent control and energy inputs are quite close in time, there will be an emergent sensation of continuity. In the words of (Gawthrop et al., 2011, p. 31): “It is shown that when event thresholds are small and sampling is regular, the intermittent controller can masquerade as the underlying continuous-time controller and thus, under these conditions, the continuous-time and intermittent controller cannot be distinguished. This explains why the intermittent control hypothesis is consistent with the continuous control hypothesis for certain experimental conditions.”

A major challenge in research on intermittency is then to detect and qualify intermittency in human body motion data (Loram et al., 2014). One solution is to look for discontinuities in the motion trajectories, something that was done already in the pioneering work of Craik and Vince several decades ago (Craik, 1947; Vince, 1948), and which has been done again recently with more developed technologies and analysis methods (Sakaguchi, 2013). There is additionally the phenomenon of the so-called *pre-motion silent period*, meaning that before the onset of a ballistic muscle contraction, there is a relaxation of the muscles that can be detected in the EMG signals (Aoki et al., 1989).

Applied to our context of sound-motion objects, we may understand music performance as concatenations of sound-motion objects, where the output may be perceived as a continuous stream, but where the control and energy input at the object timescale may be intermittent. However, this raises an important question:

If we think of intermittency as associated with salient points in time, what are then these salient point in time in music? We could mention downbeats and other salient points in time such as melodic peaks. However, it seems that downbeat is a strangely little researched topic, and one possibility could then be that it is associated with impacts, i.e., what can be seen as *velocity reversals* in a motion trajectory, something that seems to make good sense in some cases (see Godøy, 2013 for how downbeats in a waltz pattern reflects this velocity reversal). More cases of velocity reversals and other discontinuities in the motion signals could be interesting to examine, e.g., as suggested in (Sakaguchi, 2013).

In our own work, we are presently looking at discontinuities in the motion capture trajectories (e.g., the mentioned velocity reversals), mostly focused on short, rapid, and highly pre-programmed sound-motion objects in the form of ornaments and other figures. However needless to say, there are several challenges in getting good motion capture data here because we need to use a fairly large number of reflective markers and relatively high framerates in order to capture small-scale and rapid sound-producing effector motion (Song and Godøy, 2016).

### Triggers

Accepting the existence of preprogrammed motion chunks and intermittency, the next question is how these motion chunks are triggered. Unfortunately, this seems to be a not well researched topic in human motor control, yet a crucial topic for understanding the workings of skilled behavior in music performance. Assuming there is a volitional initiation of body motion in music performance, the question becomes: *What is actually the triggering mechanism in such time-sensitive tasks and how does it work?*

We have in recent years seen research on so-called entrainment in music, demonstrating that our organism picks up salient motion-inducing patterns in musical sound which in turn may result in body motion, e.g., in dancing, walking, or gesticulating (Clayton et al., 2013), and highly synchronized triggering is also documented in group performance contexts (Palmer, 2013). Yet it seems that the question still remains of what is the initial impulse to start the assumed ready-planned motion chunks.

An impulse in our context can be understood as a short burst of energy, what in physiological terms may be called a *ballistic muscle contraction*. An EMG signal with a steep attack slope could then be an indicator of a trigger, resembling a salient rhythmic articulation by the shape of the beat (Elliott et al., 2009). More generally, it could be interesting to look at the so-called *startle reactions*, as these reactions are hypothesized to work by high degrees of pre-programming activated by some loud noise or other sudden sensory impulse. It has been suggested that these triggers and the corresponding extremely fast reaction times are an integral part of our motor control system and could also be at work in more ordinary, non-startle induced motion (Valls-Solé et al., 1999, 2008).

Fast triggering of motion can be found in various sports, and an important feature here is that the triggering impulse may be rather simple, yet activate quite complex patterns of musculoskeletal motion, i.e., be part of an eminently hierarchical control scheme as suggested in (Karniel, 2013). This seems also to be the case in writing (Plamondon et al., 2013) and graffiti motion (Berio et al., 2017), research that is summarized as follows: “In our work we rely on a family of models known as the Kinematic Theory of Rapid Human Movements, mainly developed by R. Plamondon et al. in an extensive body of work since the 90’s.”… “They show that if we consider that a movement is the result of the parallel and hierarchical interaction of a large number of coupled linear systems, the impulse response of such a system to a centrally generated command asymptotically converges to a lognormal function. This assumption is attractive from a modelling perspective because it abstracts the high complexity of the neuromuscular system in charge of generating movements with a relatively simple mathematical model, which further provides state of the art reconstruction of human velocity data.” (*Ibid*, p. 2).

Lastly, some intermittency research has made a distinction between clock-based (internal) and event-based (external, adaptive) intermittent control (Sakaguchi et al., 2015), suggesting that the latter mode of triggering is more flexible and well suited to real-world demands.

The nature and workings of triggering are still a major outstanding issue, and hopefully, it will be possible to design experimental paradigms in music performance for exploring this further. Triggers are about feedforward and open loop kinds of motor control. Triggering is also about recognizing the typical sound-motion object timescale where intermittent control and energy input are optimal, as well as recognizing the possibly negative interference of input in the course of an ongoing sound-motion chunk. It seems better to leave an ongoing sound-motion object alone and let it run its course, as has been suggested in connection with chunking and the basal ganglia: “Chunks take their advantage from being manipulable as entities, and the intervention of consciousness or attention might actually disrupt their smooth implementation.” (Graybiel, 1998, p. 131).

### Sound-Motion Objects

From the various research and ideas presented so far in this paper, we can now summarize the main ideas of sound-motion objects as follows:

1. Musical features are intrinsically multimodal, comprising sensations of both sound and corresponding body motion.
2. The most salient perceptual features of both sound and motion are found at the meso timescale of approximately 0.3–5 s.
3. The intermittency hypothesis suggests that motion chunks, and the resultant sound chunks, are optimally conceived and triggered discontinuously.

Needless to say, there are several substantial challenges here:

Firstly, in understanding in more detail the interaction of sound and motion in our minds and bodies, and to have more well-informed notions of *what is what* in musical features such as rhythm and textures: What is the sound sensation and what is the motion sensation, e.g., in listening to drumming or to a string ensemble?

Secondly, there are substantial challenges in understanding the workings of timescales of both sound and body motion. The basic hypothesis here is that the meso timescale of sound-motion objects is privileged in that it encompasses the most salient sound features and the most salient motion features, i.e., that smaller timescales just do not have these features and that larger timescales do not allow the same focus on these features as was one of the main arguments for Schaeffer in favor of the sound object (Godøy, 2021).

Thirdly, there is the challenge of better understanding the initiation, or triggering, of sound producing motion. Clearly, there is preplanning going on in musical performance, but what does such a preplan look like? Is it a kind of compressed mental image of motion trajectories to be triggered? And is there some kind of ultra-rapid triggering for such preprogrammed motion chunks as suggested by the mentioned startle research?

In spite of these and similar outstanding questions, what seems to be reasonably well supported is that in music performance, there is a shaping of motion control and motion effort going on, manifest in the mentioned elements of phase transition, coarticulation, muscle synergies, idiom use, and optimization of energy use. In Western music, this shaping means transforming otherwise discrete tone events of common practice notation into coherent coarticulated sound-motion objects. The advantage of the sound-motion object idea in music performance is that of focusing on the motion optimization at the object timescale, guided by the inherent constraints of both instruments and body motion, as well as the perceptual constraints favoring holistic objects at the meso timescale. Notably so, this includes most sound features unfolding within the confines of a sound-motion object, including timbral, harmonic, and modal flavors (Persichetti, 1962), determined by the cumulative impressions of characteristic intervals over a certain time stretch.

The idea of sound-motion objects as presented in this paper is primarily concerned with motion and sound sensations, but there is no denial that such sound-motion object may have multiple significations, for instance, as is the claim of ecological acoustics (Gaver, 1993) and largely in line with ideas of sonic event perception (Rocchesso and Fontana, 2003) as well as research on auditory object perception (Bizley and Cohen, 2013). In addition, links have been made between an object focus and semiotics, as found in the UST project (Delalande et al., 1996), with the idea of “temporal semiotic units” (*unités sémiotiques temporelles*). The semiotic aspect here could also be seen as related to more semantic and/or hermeneutic aspects of sound-motion objects, needless to say important and extensive areas of music research that hopefully could be the focus of future research.

Also, there are of course features at larger timescales, i.e., the macro timescale, which could likewise be overviewed in an “all-at-once” manner, as suggested by Paul Hindemith of an entire composition: “If we cannot, in the flash of a single moment, see a composition in its absolute entirety, with every pertinent detail in its proper place, we are not genuine creators.” (Hindemith, 2000, p. 61). Zooming back and forth between such different levels of resolution is clearly a possibility, an important topic that deserves more systematic research.

## Discussion

From various available evidence, it seems reasonable to infer that sound-motion objects can play an important role in music performance, yet that the corollary notions of a fundamental discontinuity and the associated intermittency hypothesis may go against established ways of thinking. Hence, we can list here some elements that are in favor of the sound-motion object approach, followed by some elements that go against this approach, lastly followed by some ideas on how to continue exploring sound-motion objects:

- Various motion and motor control constraints seem to favor the meso timescale approach to sound-producing motion, including the intermittency hypothesis, because there is the need to work around the constraints of PRP by pre-programming.
- Sound-motion objects seem well-suited to help us understand rhythmical, textural, modal, contoural, and timbral patterns, because they provide a local context that fuse individual events into strongly coarticulated and coherent entities.
- The basically physical-physiological and motor control approach of sound-motion objects to musical features could open up for more cross-cultural assessments of musical expression.

In current music research, we have seen a focus on features of music performance such as nuances in timing and dynamics, research often using advanced measurements and processing methods and leading to interesting findings about timing-related issues. But what seems to be less focused on is the meso timescale shapes of sound-motion objects, meaning features of the object considered as a whole, as was one of the main aims of Schaeffer’s sound object theory.

But surely, there are significant unsolved problems with this sound-motion objects approach, such as the following:

- There seem to be divergent opinions on feedforward and intermittency in motor control research, and it may be that some of the ideas presented above will be contradicted by other research.
- Body motion and motor control constraints have not been much focused on in mainstream music research; hence, the idea of including various constraints in the analysis of music may require new ways of thinking in music-related research.
- The view of music as a series of sound-motion objects may go against the idea of music as a continuous flow, yet the concept of sound-motion objects could be well reconciled with the ideas of music as continuous sensory streams, provided we have an awareness of the different timescales simultaneously at work here.
- There are substantial challenges in collecting more precise motion capture data and more extensive EMG data on effort distribution in support of the intermittency hypothesis.

As for further work, there are three main areas that we are hoping to work in:

- *Conceptual-analytic work*: Clearly, there are inherited issues in music theory and also in performance research that may need critical assessment, in particular in view of how meso timescale salient sound and body motion features are handled. Said differently, there seems to be a lack of object focus in mainstream music analytic thinking, hence the need to exercise shifts of timescale perspective, i.e., ask questions about what we are focusing on, as was the strategy of Schaeffer, and then going on to represent salient features as holistic shapes. Also in terms of motor control, there seems to be a need for more critical assessment of inherited control theory, in particular in view of how meso timescale object-focused features are taken care of in the control schemes.
- *Exploratory modeling*: Given the difficulties in detecting intermittency in motor control, it could be useful with a reverse engineering approach of simulating impulse-driven motion chunks by combining pre-programmed shapes with intermittent, point-by-point energy input. This could be a heuristic strategy to discover what would be the workings and requirements in real-life intermittent control situations for sound-producing motion. We are presently making some simplified toy models of intermittency, inspired by so-called impulse-response modeling, i.e., basically by having an energizing impulse that dissipates its energy through a stationary shape by convolution.
- *Testable hypotheses*: This is about formulating various sound-producing motion tasks for performers that may be carried out either intermittently or continuously, and assessing the results. We envisage using a combination of motion capture and EMG to record both the output motion and the muscular effort that go into the motion. Participants will concretely be asked to move as abruptly as possible (i.e., ballistic motion) followed by relaxation phases, or as smoothly as possible (i.e., fluency) with continuous effort, hopefully providing insights into intermittent vs. more continuous energy input in sound-producing motion.

As for possible practical applications of ideas of constraint-based sound-motion objects, the following could be suitable exploratory schemes in cooperation with musicians:

- In instrument practice experiments, the phenomena of phase transition and coarticulation could be explored by alternating between very different tempi, enabling more direct observations of the effects of tempi on sound-motion object cohesion and other emergent features.
- In improvisation, and by extension also in composition, work specifically with sound-motion objects as combined motor gestalts and sound objects, focusing on the overall typological shapes and sense of motion, reminiscent of David Sudnow’s book on motion-based improvisation (Sudnow, 1978).

In summary, the core idea of this paper is due to an object perspective in music, originally proposed by Pierre Schaeffer and applied to the *musique concrète*, then successively to other kinds of musical sound, and now also to body motion with the concept of sound-motion objects. The motivation for all this is the belief that sound-motion objects are optimal for both the generation and the perception of music and could also be the source of novel analytic and creative tools.

## Data Availability Statement

The data analyzed in this study is subject to the following licenses/restrictions: The dataset is available upon application to RITMO for research purposes. Requests to access these datasets should be directed to RG, r.i.godoy@imv.uio.no.

## Author Contributions

The author confirms being the sole contributor of this work and has approved it for publication.

## Funding

The work on this paper has been partially supported by the Research Council of Norway through its Centres of Excellence scheme, project number 262762 and by the University of Oslo.

## Conflict of Interest

The author declares that the research was conducted in the absence of any commercial or financial relationships that could be construed as a potential conflict of interest.

## Publisher’s Note

All claims expressed in this article are solely those of the authors and do not necessarily represent those of their affiliated organizations, or those of the publisher, the editors and the reviewers. Any product that may be evaluated in this article, or claim that may be made by its manufacturer, is not guaranteed or endorsed by the publisher.
